# Intra-Uterine Dropsy

**Published:** 1847-06

**Authors:** 


*Intra-Uterine Dropsy.*—At a late meeting of Bath and Bristol Branch of the Provincial Medical and Surgical Association, Mr. King narrated a case of dropsy occurring in a fœtus of eight months. The mother had borne six children, but did not menstruate between her first and fifth pregnancies; was "unwell" once when she became pregnant with this child. Between the fifth and sixth months she became anasarcous, and, at the time of parturition, her abdomen and lower extremities were very large, tense, and hard. Some few days after labour her urine was albuminous. The labour was natural, but the child did not advance, though the pelvis was large, and the head of the child could be freely moved therein. The uterus was excited to action by two doses of ergot, and the child, with some difficulty, caused by the size of its body, brought down. At birth it measured round the waist sixteen inches and a half.—*Provincial Medical and Surgical Journal.*

Remarks.—We have cited this case on account of its interest in an obstetric point of view; otherwise the absence of any notice of a cadaveric inspection renders it very unsatisfactory. An examination of the thoracic and abdominal viscera, and especially of the heart and kidneys, might have given rise to some important pathological results.—*London Medical Gazette.*

